# Comprehensive genomic analysis of adrenocortical carcinoma reveals genetic profiles associated with patient survival

**DOI:** 10.1016/j.esmoop.2024.103617

**Published:** 2024-06-26

**Authors:** A. Sun-Zhang, C.C. Juhlin, T. Carling, U. Scholl, M. Schott, C. Larsson, S. Bajalica-Lagercrantz

**Affiliations:** 1 Department of Oncology-Pathology, Karolinska Institutet, Stockholm; 2 Department of Pathology and Cancer Diagnostics, Karolinska University Hospital, Stockholm, Sweden; 3 Carling Adrenal Center & Hospital for Endocrine Surgery, Tampa; 4 Yale Endocrine Neoplasia Laboratory, Yale School of Medicine, New Haven, USA; 5 Center of Functional Genomics, Berlin Institute of Health at Charité – Universitätsmedizin Berlin, Berlin; 6 Division for Specific Endocrinology, Medical Faculty, University Hospital Düsseldorf, Heinrich-Heine-University, Düsseldorf, Germany; 7 Department of Clinical Genetics, Karolinska University Hospital, Stockholm, Sweden

**Keywords:** adrenocortical carcinoma, mRNA, mutation, protein–protein interaction, survival

## Abstract

**Background:**

Adrenocortical carcinoma (ACC) is one of the most lethal endocrine malignancies and there is a lack of clinically useful markers for prognosis and patient stratification. Therefore our aim was to identify clinical and genetic markers that predict outcome in patients with ACC.

**Methods:**

Clinical and genetic data from a total of 162 patients with ACC were analyzed by combining an independent cohort consisting of tumors from Yale School of Medicine, Karolinska Institutet, and Düsseldorf University (YKD) with two public databases [The Cancer Genome Atlas (TCGA) and Gene Expression Omnibus (GEO)]. We used a novel bioinformatical pipeline combining differential expression and messenger RNA (mRNA)- and DNA-dependent survival. Data included reanalysis of previously conducted whole-exome sequencing (WES) for the YKD cohort, WES and RNA data for the TCGA cohort, and RNA data for the GEO cohort.

**Results:**

We identified 3903 significant differentially expressed genes when comparing ACC and adrenocortical adenoma, and the mRNA expression levels of 461/3903 genes significantly impacted survival. Subsequent analysis revealed 45 of these genes to be mutated in patients with significantly worse survival. The relationship was significant even after adjusting for stage and age. Protein–protein interaction showed previously unexplored interactions among many of the 45 proteins, including the cancer-related proteins DNA polymerase delta 1 (POLD1)*,* aurora kinase A (AURKA), and kinesin family member 23 (KIF23). Furthermore 14 of the proteins had significant interactions with TP53 which is the most frequently mutated gene in the germline of patients with ACC.

**Conclusions:**

Using a multiparameter approach, we identified 45 genes that significantly influenced survival. Notably, many of these genes have protein interactions not previously implicated in ACC. These findings may lay the foundation for improved prognostication and future targeted therapies.

## Highlights

• A 45-gene signature for ACC was constructed using a multi-omics pipeline.
• Patients with the 45-gene signature had significantly worse prognosis even after adjusting for stage and age.
• Protein–protein interaction analysis revealed interactions not previously implicated in ACC.
• Several genes in the signature have therapeutic potential as targets or markers.

## Graphical abstract

**Figure:**
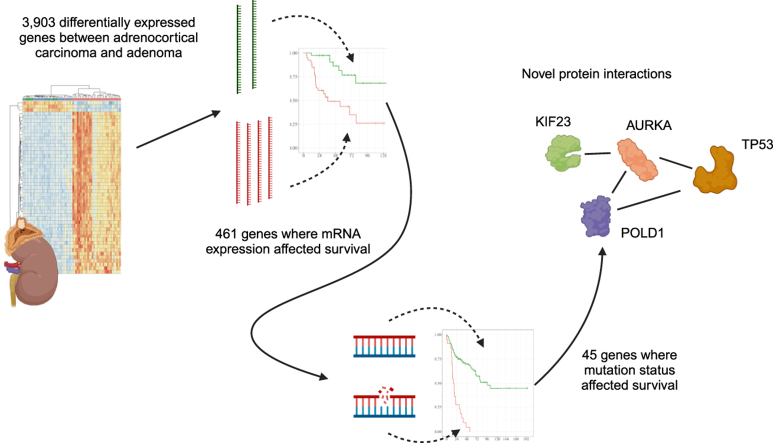

## Introduction

Adrenocortical carcinoma (ACC) is a rare form of cancer with a worldwide incidence between 1 and 2 per million per year. It is one of the deadliest endocrine malignancies, with a median overall survival of ∼3-4 years. The median survival for patients with advanced disease is ∼1 year with 5-year survival rates between 0% and 28%.^1^, ^2^, ^3^ The median age at presentation is ∼50 years with predominantly females being affected.^2^^,^^4^ More than one-half of ACCs are hormone producing, with the two most common hormone profiles being hypercortisolism and excessive androgen secretion.^5^ At present, surgical tumor resection is the only curative treatment for localized disease and brings the best possibility for prolonged survival in advanced disease; however, despite its availability, disease recurrence is reported in ∼60%-70% of patients.^6^^,^^7^ To date, mitotane is the only antineoplastic agent approved by the European Medicines Agency (EMA) for the treatment of ACC, but clinical efficacy of the drug varies, with several studies reporting conflicting results in addition to its severe side-effects.^5^^,^^8^^,^^9^

Although most ACCs arise sporadically through the acquisition of somatic genetic aberrations, subsets of ACC can be caused by germline alterations in genes predisposing for hereditary cancer risk syndromes such as Li–Fraumeni syndrome, Beckwith–Wiedemann syndrome, multiple endocrine neoplasia type 1, and Lynch syndrome. Interestingly, 50%-80% of all children with ACC and up to 4% of all patients with ACC, regardless of age, have germline mutations in *TP53*.^10^, ^11^, ^12^, ^13^, ^14^ Numerous genes and pathways have been demonstrated to be frequently dysregulated in sporadic ACC. These include *CTNNB1*-activating mutations and *ZNRF3* deletions and mutations, both of which are pivotal mediators in the Wnt/β-catenin pathway. Overexpression of *IGF2* together with its hosted *miR-483* and downregulation of *H19* with its hosted *miR-675* are frequent findings in ACC.^15^ Moreover, *TP53* mutations are recurrently seen.^2^^,^^16^ Furthermore, genomic studies have shown that ACCs can be classified into three distinct classes based on DNA-methylation patterns which impacts genomic and clinical profile.^17^^,^^18^

Although advancements have been made in elucidating the molecular mechanisms and pathways that underlie the pathogenesis of ACC, the translation of this knowledge into the realm of clinical therapeutics, with a focus on delivering effective interventions for patients, has been limited. Furthermore, there is still a notable absence of significant genetic profiles and lack of genetic markers for the diagnosis and prognosis as the link between genotype and phenotype leaves much to be explored. Therefore there is a critical need for further identification of genetic markers that are related to the diagnosis and clinical outcome in patients with ACC.^2^^,^^5^^,^^11^

This study combines independent genetic and clinical data from Yale School of Medicine, Karolinska Institutet, and Düsseldorf University with public data from The Cancer Genome Atlas (TCGA) and the Gene Expression Omnibus (GEO). Our objective was to discover and characterize ACC-specific genetic signatures that significantly influence the prognosis of patients.

## Materials and methods

### The Yale–Karolinska–Düsseldorf (YKD) cohort

In total, whole-exome sequencing (WES) data were reanalyzed from 41 primary sporadic ACC cases previously recruited from three different institutions: Yale School of Medicine, New Haven, USA (*n* = 19); Karolinska University Hospital, Stockholm, Sweden (*n* = 14); and Düsseldorf University, Düsseldorf, Germany (*n* = 8), referred to as the Yale–Karolinska–Düsseldorf (YKD) cohort.^19^ The data exclusively rely on somatic variants, following a prior filtering of constitutional variants. None of the patients had undergone neoadjuvant chemotherapy nor radiotherapy at the time of tissue collection. Yale and Düsseldorf samples were formalin-fixed, paraffin-embedded, whereas samples from Karolinska were fresh-frozen. Tumor and normal adrenal tissue (NAT) purity was examined by endocrine pathologists for all samples before extraction of genomic DNA and creation of DNA libraries. WES was previously carried out using the Illumina platform and sequences were aligned to NCBI Build 36 of the human genome.^19^ The mean number of reads did not differ significantly between formalin-fixed, paraffin-embedded and fresh-frozen tissues. For this study, WES data from all 41 cases were used; however, 4 samples without survival date were removed from further downstream analysis (described later).

### Public ACC cohorts

WES (somatic mutations), RNA sequencing, and clinical ACC data were retrieved from TCGA through the National Cancer Institute (NCI) application programming interface using the R package ‘TCGAbiolinks’ (R Foundation, Vienna, Austria).^17^ Data were retrieved on September 6, 2023 and all 92 patients with ACC were included for analysis. Only one patient received neoadjuvant treatment before surgery. All analyzed tumors were primary. Transcriptomic data based on Affymetrix arrays and clinical data on an additional 33 ACC cases were downloaded from GEO (GSE10927) using the R package ‘GEOquery’. The GEO cohort also included 22 adrenocortical adenomas (ACA) and 10 samples from NAT.^20^

### Differentially expressed genes

Differentially expressed genes (DEGs) between ACC, ACA, and NAT were identified in the GEO data using the R package ‘limma’. Expression data were converted into base 2 log from base 10 log. Expression data were weighted using restricted maximum likelihood scoring iterations to adjust for expression variability. The *P* values were adjusted using the Benjamini–Hochberg procedure. The fold-change significance threshold was set at 1.5 (logFC = 0.58). Heatmaps and clustering were generated using the R package ‘pheatmap’.

### Hypergeometric enrichment and gene set enrichment analysis

Hypergeometric enrichment analysis and gene set enrichment analysis (GSEA) were carried out using the R packages ‘clusterProfiler’ and ‘fgsea’. Gene sets were retrieved from the Broad Institute Molecular Signatures Database (https://www.gsea-msigdb.org/gsea/msigdb), either manually or using the R package ‘msigdbr’. Hallmark gene sets (H) and oncogenic signature gens sets (C6) were used in the analysis. All DEGs were analyzed using GSEA, and only significantly different genes were used for hypergeometric enrichment analysis. *P* values were adjusted for multiple testing using the Benjamini–Hochberg procedure.

### Protein–protein interaction analysis

Protein–protein interactions (PPIs) were analyzed using the STRING database version 12.0 (June 23, 2023).^21^ The included interaction sources were text mining, experiments, databases, co-expression, neighborhood, gene fusion, and co-occurrence. The minimum interaction score was set at 0.4.

### Statistics and visualization

All analyses and visualization, unless otherwise specified, were carried out using R version 4.2.2. Significant threshold was set at an adjusted *P* < 0.05. Fisher’s exact test and Pearson’s chi-square test were used to analyze the independency of categorical measurements, Kruskal–Wallis one-way analysis of variance for analyzing differences in the median value between two or more groups, and Welch two-sample *t*-test for evaluating the difference in mean between two groups. Survival analysis was conducted using the log-rank test or multivariate Cox regression adjusting for stage and age and visualized using the R packages ‘survival’ and ‘survminer’. Survival was presented as overall survival and defined as the time from the date of tumor diagnosis to death of any cause. For the YKD cohort, cancer-specific survival was used to confirm that all patients harboring the gene signature died from cancer-specific causes. Messenger RNA (mRNA) data from TCGA were analyzed using the R package ‘DESeq2’. A Weiss score above or equal to the median was defined as high, and a Weiss score lower than the median was defined as low. mRNA expression strata was defined using the median expression, and tumor mutational burden (TMB) strata was similarly defined using the median mutation burden. *P* values for mRNA survival data were adjusted for multiple testing using the Benjamini–Hochberg procedure. Somatic mutation data from the YKD cohort and TCGA were converted into mutation annotation files (maf) and analyzed and visualized using the R package ‘maftools’. All figures, unless otherwise specified, were created using the R package ‘ggplot2’ with certain labels added in Microsoft PowerPoint (Redmond, WA).

### Study approval

The use of all the patient material was approved by the Yale University Institutional Review Board, the Swedish Ethical Review Authority, and the German Ethical Review Authority after obtaining informed consent from all patients.

## Results

### Effects of clinicopathological characteristics on ACC survival

No significant differences regarding the main clinical characteristics sex, stage, metastases, or Weiss score were observed between the YKD, TCGA, and GEO cohorts (Supplementary Table S1, available at https://doi.org/10.1016/j.esmoop.2024.103617). There were more females than males in all cohorts (YKD: *P* = 0.033; TCGA: *P* = 0.0035; and GEO: *P* = 0.024), with an overall ratio of 2 : 1. The mean age at tumor diagnosis was 49 (range 14-87) years. Among all cohorts, 54% of patients had a stage I-II disease at diagnosis and 46% had a stage III-IV disease. In the TCGA cohort, 51% of patients had a high (above or equal to the median value in the TCGA dataset) Weiss score (≥6).

Overall survival was analyzed in all cohorts combined (Supplementary Table S1; Supplementary Figure S1, available at https://doi.org/10.1016/j.esmoop.2024.103617) and adjusted for tumor stage and age. A higher tumor stage was associated with worse patient survival (*P* < 0.0001), with one-half of all patients with stage IV disease dead at 17.9 months; by contrast, all patients with stage I disease were still alive (Supplementary Figure S1A and B, available at https://doi.org/10.1016/j.esmoop.2024.103617). Sex, age at diagnosis, and Weiss score were not associated with survival (sex: *P* = 0.54; age at diagnosis: 0.22; and Weiss score: *P* = 0.12; Supplementary Table S1, available at https://doi.org/10.1016/j.esmoop.2024.103617).

Hormone profiles for 36 patients (97%) in the YKD cohort and 8 patients (33%) in the GEO cohort are described in Supplementary Table S2, available at https://doi.org/10.1016/j.esmoop.2024.103617, but were not available for the TCGA cohort. Patients with cortisol-producing tumors had worse survival compared with those without cortisol production (median survival 26.8 months versus 96.7 months; *P* = 0.023, Supplementary Figure S1C, available at https://doi.org/10.1016/j.esmoop.2024.103617, adjusted for stage and age: 0.016).

### Differentially expressed genes between NAT, ACA, and ACC

**Figure 1:**
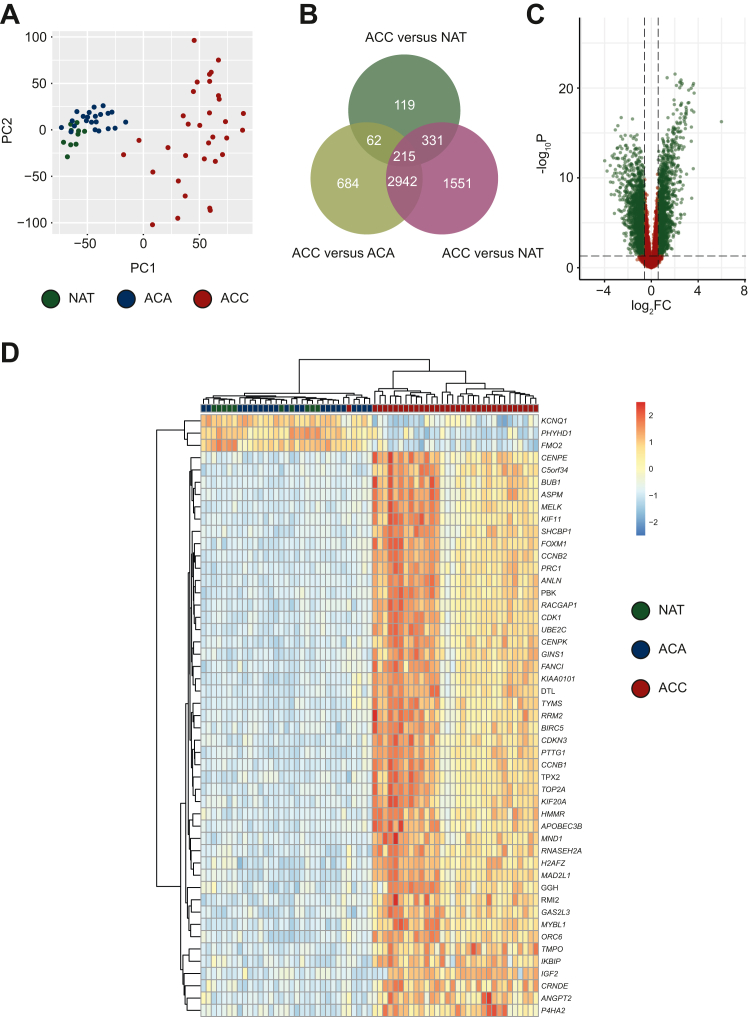
**Messenger RNA (mRNA) expression in normal adrenal tissue (NAT), adrenocortical adenoma (ACA), and carcinoma.** Differentially expressed genes were identified from transcriptomic data for the Gene Expression Omnibus (GEO) cohort of NAT (*n* = 10), ACA (*n* = 22), and adrenocortical carcinomas (ACCs; *n* = 33). (A) ACCs plotted against principal component one (PC1) and two (PC2) based on mRNA expression. ACCs showed a higher intracluster heterogeneity and also clustered further away compared with ACAs and NAT. (B) Venn diagram showing the overlap between the three sets of differentially expressed genes: ACA versus NAT, ACC versus NAT, and ACC versus ACA. (C) Volcano plot showing all differentially expressed genes between ACC and ACA with samples *P* < 0.05 and fold change >1.5 in green and all other samples in red. (D) The 50 most differentially expressed genes based on *P* value between ACC and ACA, clustered by expression similarity. FC, fold change.

DEGs were identified in the GEO cohort by comparing transcriptomic data for ACC, ACA, and NAT. Principal component analysis revealed close clustering of ACA and NAT, suggesting similar transcriptomic signatures. ACCs clustered further away from ACA and NAT, suggesting distinct expression patterns. ACCs also had a higher intracluster heterogeneity compared with ACA and NAT (Figure 1A and Supplementary Figure S2, available at https://doi.org/10.1016/j.esmoop.2024.103617).

There were 3903 DEGs between ACC and ACA (Figure 1B and C). A heatmap was constructed (Figure 1D) using the top 50 most DEGs (based on *P* value), including 47 upregulated and 3 downregulated transcripts in ACC compared with ACA (Supplementary Table S3, available at https://doi.org/10.1016/j.esmoop.2024.103617). In the heatmap, NAT and ACA showed similar expression profiles, which differed from that of ACC.

### Association between mRNA expression levels and survival

**Figure 2:**
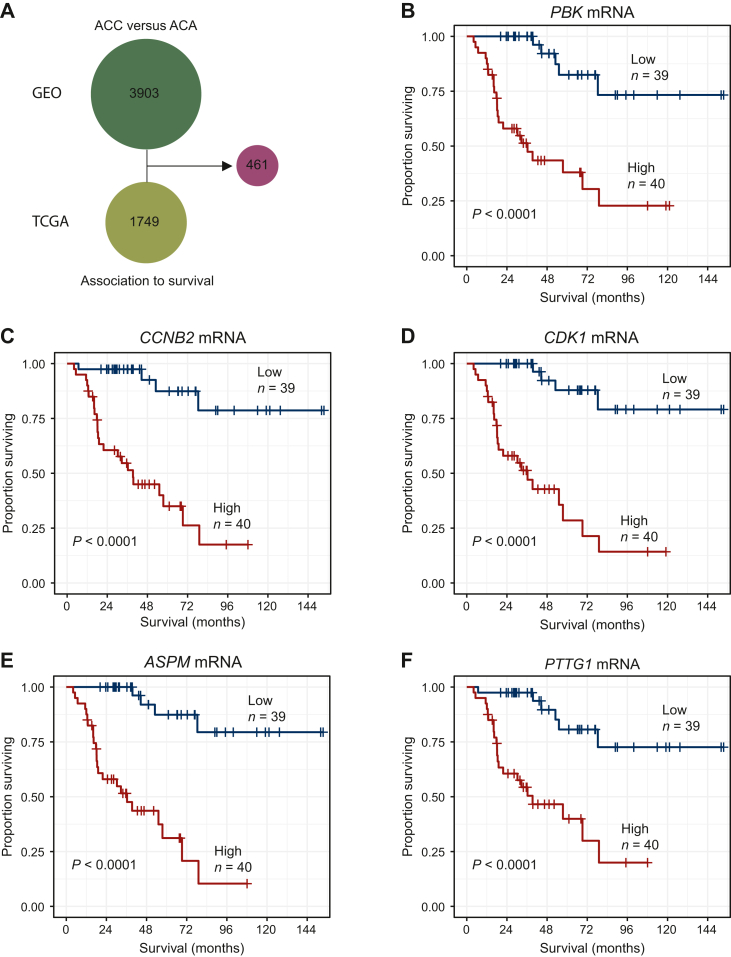
**Association between messenger RNA (mRNA) expression and overall survival for differentially expressed genes.** (A) Schematic illustration of identification of 461 genes associated with survival in The Cancer Genome Atlas (TCGA) cohort and differentially expressed between adrenocortical carcinoma (ACC) and adrenocortical adenoma (ACA) in the Gene Expression Omnibus (GEO) cohort. (B–F) Kaplan–Meier survival curves for ACC cases from the TCGA cohort for high and low mRNA expression levels determined as above or below the median. High mRNA expression of (B) PBK (PDZ-binding kinase); (C) CCNB2 (G2/mitotic-specific cyclin-B2); (D) CDK1 (cyclin-dependent kinase 1); (E) APSM (assembly factor for spindle microtubules); and (F) PTTG1 (PTTG1 regulator of sister chromatid separation) was associated with worse survival.

Analysis of overall survival was carried out in ACC cases from the TCGA cohort. All tumors analyzed were primary. In total, the expression levels of 1749 mRNA transcripts were significantly associated with overall survival. Of these transcripts, 461 (26%) were significantly differentially expressed when comparing ACC and ACA (Figure 2A; Supplementary Table S4, available at https://doi.org/10.1016/j.esmoop.2024.103617). Multivariate Cox regression showed 388/461 transcripts were independently associated with survival, even after adjusting for tumor stage and age. The five transcripts with the highest absolute fold changes and with impact on survival are illustrated in Figure 2. These were *PBK*, *CCNB2*, *CDK1*, *ASPM*, and *PTTG1*. For all these genes, higher mRNA expression was associated with worse survival (*P* < 0.0001; Figure 2B-F).

### Hypergeometric enrichment and gene set enrichment analysis

GSEA of all 3903 DEGs comparing ACC and ACA in the GEO cohort revealed that 35 hallmark (H) pathways were affected, including 13 upregulated and 22 downregulated pathways (Supplementary Table S5, available at https://doi.org/10.1016/j.esmoop.2024.103617). The most upregulated pathway was ‘E2F TARGETS’, which corresponds to genes encoding for cell cycle-related targets of E2F transcription factors. Hypergeometric enrichment analysis showed that 78% of all genes in the ‘E2F TARGETS’ pathway were also significantly differentially expressed when comparing ACC and ACA. GSEA was also evaluated using the oncogenic signature gene set (C6). This identified 45 enriched pathways, of which 26 were upregulated and 19 were downregulated (Supplementary Table S5, available at https://doi.org/10.1016/j.esmoop.2024.103617). The most upregulated pathway was ‘RB P107 DN.V1 UP’ which corresponds to upregulated genes in response to *RB1* knockout. Hypergeometric enrichment analysis showed that 68% of all genes in the ‘RB P107 DN.V1 UP’ pathway were significantly differentially expressed when comparing ACC and ACA. GSEA was then carried out for the genes that were both differentially expressed between ACC and ACA and where mRNA expression level was associated with survival (Supplementary Table S5, available at https://doi.org/10.1016/j.esmoop.2024.103617). This revealed significant enrichment in four hallmark pathways, all of which were upregulated. The most upregulated pathway was also ‘E2F TARGETS’. In addition, enrichment was observed in six oncogenic signature C6 pathways, which were all upregulated (Supplementary Table S5, available at https://doi.org/10.1016/j.esmoop.2024.103617).

### Effects of tumor mutational burden on tumor characteristics and survival

**Figure 3:**
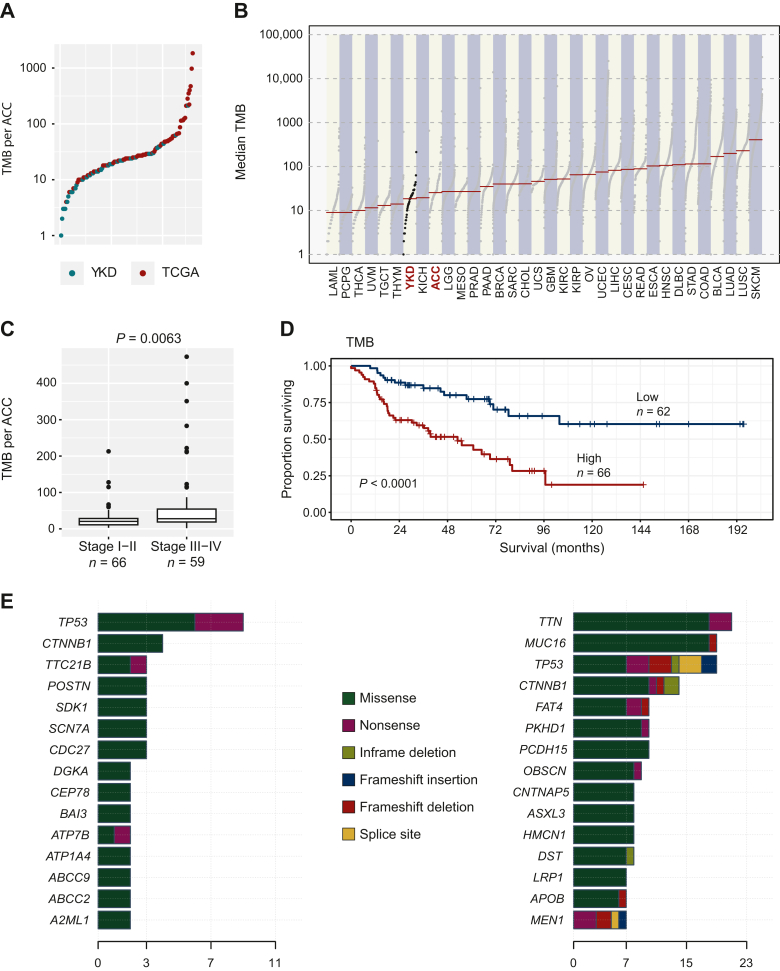
**Tumor mutational burden (TMB).** (A) Scatterplot of TMB for each adrenocortical carcinoma (ACC) sample from the Yale–Karolinska–Düsseldorf (YKD; teal) and The Cancer Genome Atlas (TCGA; red) cohorts showing similar levels in the two cohorts. (B) Comparison of median TMB across all TCGA cancer sets with integration of the YKD cohort. The YKD and TCGA–ACC cohorts are indicated. The median TMB of the YKD cohort was significantly lower compared with four of the TCGA cohorts: uterine corpus endometrial carcinoma (UCEC, *P* < 0.0001), melanoma (SKCM, *P* < 0.0001), colon adenocarcinoma (COAD, *P* = 0.0094), and stomach adenocarcinoma (STAD, *P* = 0.031). The same four cohorts had significantly higher median TMB compared with TCGA–ACC (UCEC, *P* < 0.0001; SKCM, *P* < 2 0.0001; COAD, *P* = 0.0010; STAD, *P* = 0.0077). (C) Boxplot illustrating higher TMB in stage III-IV tumors than in stage I-II tumors in the YKD and TCGA cohorts combined. Sample with the highest TMB removed from analysis (TMB 1841). (D) A high TMB was associated with worse survival even after adjusting for stage. (E) The top 15 genes with most total mutations and corresponding mutation type in the YKD cohort (left) and the TCGA cohort (right), respectively. The bars correspond to the number of mutations detected.

TMB, defined as the total number of coding mutations per tumor case, was investigated using WES data for the YKD and TCGA cohorts. The median TMB for the YKD cohort was 18.5 (range 1-213) and 27 (range 4-1841) for the TCGA cohort. The median TMB did not differ significantly between the two cohorts (*P* = 0.91; Figure 3A). The median TMB in the YKD and TCGA cohorts were compared across all TCGA cohorts, of which melanoma (SKCM cohort) was the tumor type with the highest median TMB (median TMB 4065) and acute myeloid leukemia (LAML cohort) and pheochromocytoma and paraganglioma (PCPG cohort) were the two tumor cohorts with the lowest median TMB (median TMB 9). The YKD and TCGA-ACC cohorts were the seventh and ninth least mutated cohorts (Figure 3B).

TMB in individual ACCs was compared with clinical characteristics and survival. This revealed a higher TMB in stage III-IV ACCs compared with stage I-II disease (mean TMB 66.24 versus 28.35, *P* = 0.0063; Figure 3C). A trend toward higher TMB was observed for ACCs with high as compared with low Weiss score (mean 77.59 versus 36.41; *P* = 0.064). Patients with a high TMB had worse survival compared with patients with a low TMB (*P* < 0.0001; Figure 3D). Furthermore, the survival difference persisted even after correcting for stage and age, suggesting TMB as an independent prognostic factor regardless of stage and age (*P* = 0.022).

The top 15 genes with the highest numbers of total mutations and corresponding mutation types in the YKD and TCGA cohorts are shown in Figure 3E. In the YKD cohort, the highest number of coding mutations were observed in *TP53* and *CTNNB1*. In the TCGA cohort, *TTN* and *MUC16* had the highest number of coding variants followed by *TP53* and *CTNNB1*.

### The 45-gene signature

**Figure 4:**
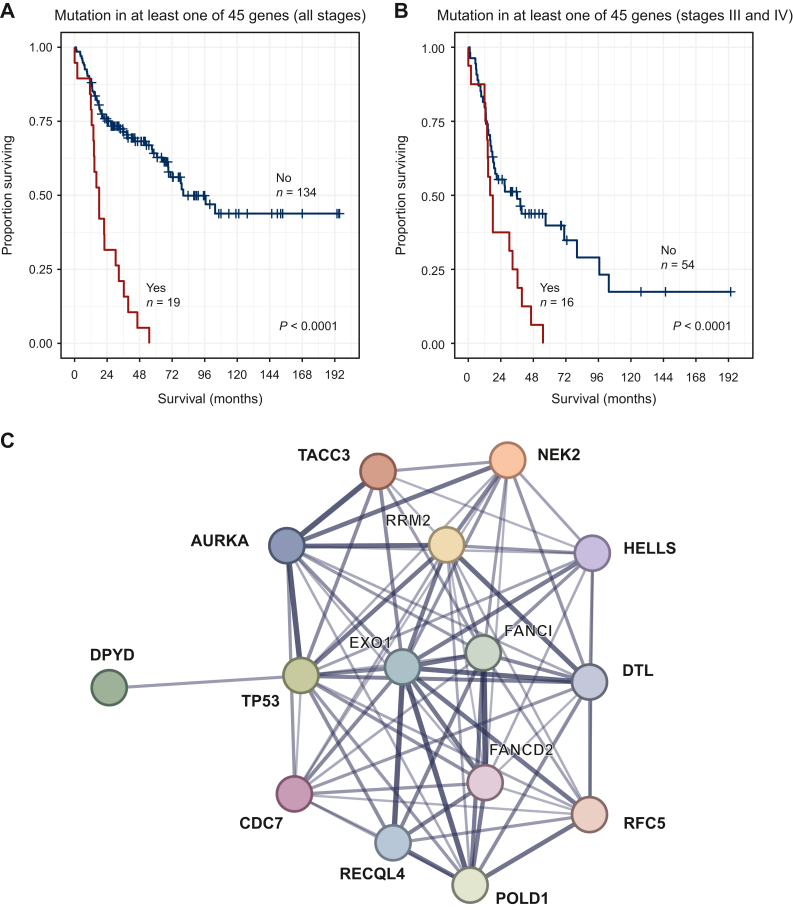
**Survival and protein–protein interaction (PPI) of the 45-gene signature.** (A) Survival curve showing significantly worse survival for patients carrying a mutation in at least one of the genes in the 45-gene signature for patients of all stages and (B) only for the patients with stage III or stage IV disease. (C) PPI analysis of the 45 genes adding *TP53* as an additional node showed 14 significant interactions with TP53.

Among the 461 genes that were both significantly differentially expressed between ACC and ACA and where mRNA expression impacted overall survival, 138 had at least one mutation in the YKD or TCGA cohort. Among those 138 genes, there were 56 genes where all mutation carriers were deceased at last follow-up. Each patient with available disease-specific data died as a result of tumor recurrence. After adjusting for stage and age using multivariate Cox regression with expression, stage, and age as covariates and adjusting for multiple testing, 45 genes affected survival independent of stage and age. The genes in this 45-gene signature are detailed in Supplementary Table S6, available at https://doi.org/10.1016/j.esmoop.2024.103617. One or more genes in the 45-gene signature were found mutated in 19 different patients from the YKD and TCGA cohorts (Table 1). The median survival time for these 19 patients was only 18.1 months, as compared with 80.2 months for all other patients (Figure 4A; *P* < 0.0001). The 45-gene signature significantly affected survival even after adjusting for stage and age using Cox multivariate regression (*P* = 0.0017) and the difference in survival also persisted after stratifying by stage and analyzing patients with advanced disease (stage III and stage IV) separately (Figure 4B; *P* < 0.0001).

**Table 1:** Clinical data for all patients carrying mutations in one or more of the genes in the 45-gene signature

TCGA/YKD ID	Sex	Age at diagnosis (years)	Location	Tumor stage	Survival (months)	Mutated genes in the 45-gene signature
A5OH	F	59	R	IV	0	*EML1, RFC5*
548	M	49	NA	IV	2	*TMEM150C*
A5K9	F	61	R	II	11	*C7, DTL, FANCI, KIF2C, RRM2*
A5J5	M	30	R	III	12	*ARTN, ETV5, REQL4, SLC9A9*
A5OG	F	45	L	IV	13	*CHAF1B*
A5J4	F	23	R	IV	14	*SH3D19, DPYD*
A5LI	F	42	R	IV	14	*KIF23*
A5LF	F	74	L	III	15	*GAS2L3*
A5J7	F	30	R	III	16	*GAS2L3, LTBP1*
A5JB	M	52	R	IV	18	*ALDH5A1, DIAPH3, POLD1*
A5JY	F	68	R	IV	18	*HOOK1*
A5LE	M	14	L	II	22	*MCEE*
546	F	59	NA^c^	II	22	*PPM1L*
A5JA	F	53	R	IV	30	*AURKA, BMP1, CCDC150, EXO1, HTRA3, PHF19, POLQ, SDC4, SLC7A14, TACC3, USP53*
A5K2	F	32	R	III	33	*CDC7*
A5LJ	F	54	L	IV	36	*CDCA7, HELLS, KNTC1, RNF44*
A5LB	M	59	R	IV	40	*IQGAP3, PHACTR2, PNLIPRP3, DPYD*
505	F	62	NA^c^	III	46	*FANCD2*
A5J2	F	44	L	IV	55	*NEK2*

F, female; L, left adrenal gland; M, male; NA, not available; R, right adrenal gland; TCGA, The Cancer Genome Atlas; YKD, Yale–Karolinska–Düsseldorf.

### Protein–protein interaction analysis and pathway enrichment

The PPI analysis of the 45-gene signature showed 146 total edges, while the expected number of edges by chance was 15 (*P* < 0.0001), suggesting enrichment. All interactions and interaction scores are outlined in Supplementary Table S7, available at https://doi.org/10.1016/j.esmoop.2024.103617. Several novel interactions, not previously implicated in ACC, were observed, including DNA polymerase delta 1 (POLD1), aurora kinase A (AURKA) and kinesin family member 23 (KIF23). The two most significantly dysregulated pathways in the protein network were cell cycle- and DNA-related pathways (Supplementary Table S8, available at https://doi.org/10.1016/j.esmoop.2024.103617). As *TP53* is well known to be the most frequently mutated gene in the germline of patients with ACC, we further analyzed PPI with TP53 as one of the target nodes. TP53 was then found to have 14 significant interactions (Figure 4C, Supplementary Table S9, available at https://doi.org/10.1016/j.esmoop.2024.103617). Interestingly, both AURKA (interaction score 0.998) and POLD1 (interaction score 0.592) had medium to strong interactions with TP53.

## Discussion

In this study, we identified clinical and molecular characteristics associated with poor survival, such as cortisol production and high TMB. We also identified DEGs between ACC and ACA and analyzed altered pathways using GSEA. More interestingly, we created a novel pipeline combining differential expression between ACC and ACA, the association between mRNA expression and survival, and the association between DNA mutations and survival to find 45 genes to be strongly associated with patient survival.

A pathway analysis of the DEGs showed dysregulation in cell cycle-regulating pathways such as ‘E2F TARGETS’ and ‘G2M CHECKPOINT’. These are commonly dysregulated in dysfunctional Wnt/β-catenin signaling which has previously been shown to be associated with ACC.^22^, ^23^, ^24^

Many of the key 45 genes found in this study have previously not been reported to impact clinical characteristics or outcome in ACC.^14^^,^^17^ The PPIs among these 45 genes showed a very strong interaction between KIF23 and AURKA (interaction score = 0.975), both of which are important regulators of mitosis.^25^^,^^26^ None of the previous studies have examined the survival impact of KIF23 in ACC, although several studies have demonstrated it to be a very promising negative prognostic marker in other solid tumors such as hepatocellular carcinoma and lung cancer.^26^^,^^27^ A smaller study has previously suggested AURKA to be a potential prognostic marker for ACC, and several larger studies have shown its prognostic significance for other malignancies.^25^^,^^28^, ^29^, ^30^ AURKA was also shown to interact with POLD1, an important protein for DNA repair and replication. Overexpression of POLD1 has previously been linked to poor prognosis in hepatocellular and breast cancer; however, no study has previously related POLD1 expression to survival in ACC.^31^^,^^32^ In addition, 13 interactions were observed between the 45-gene signature and TP53 including both AURKA and POLD1 (Figure 4C, Supplementary Table S9, available at https://doi.org/10.1016/j.esmoop.2024.103617). *TP53* germline mutation is the most common germline event in ACC and is observed in up to 4% of all cases and up to 80% of all pediatric cases.^33^ Studies have shown that TP53 is one of the most important substrates for AURKA and phosphorylation can lead to destabilization with deregulation of the cell cycle and oncogenic transformation.^30^^,^^34^ AURKA inhibitors have been able to sensitize tumors to radiotherapy, with effects especially prominent in tumors with *TP53* mutations.^35^ This could potentially open up new avenues for treatment in the future as radiotherapy is an important treatment option for metastasized ACC.^36^ Furthermore, a recent study published in *JAMA Oncology* has associated POLD1 dysregulation with improved immunotherapy response and an ongoing phase II trial is examining immunotherapy as monotherapy against POLD1-mutated patients with solid tumors (NCT03810339).^37^ This finding may be particularly intriguing for ACCs, where immune checkpoint inhibition has shown some, but not dramatic effects.^38^

The many significant interactions among the 45 genes could suggest previously unexplored mechanisms and pathways that together with the high frequency of germline TP53 mutations impact the clinical outcome of patients with ACC. Most importantly, many of the interacting proteins are promising targets and markers for oncological therapy.

We believe this to be the first study to combine mRNA differential expression data between ACC and ACA with both mRNA-dependent survival data and DNA mutation data to identify and characterize new genes that might affect clinical characteristics and survival in ACC. We propose that the 45-gene signature might be related to both tumor progression from ACA to ACC and most importantly patient outcomes.

One limitation of this study is the relatively small cohort size, as ACC is a rare disease. Moreover, there is a need for further research to explore the interactions and mechanisms behind the 45 genes found in this study, to better understand how they impact tumor progression and patient survival.

In conclusion, the combination of the three ACC cohorts (YKD, TCGA, and GEO) has allowed for a comprehensive evaluation of genetic alterations and signatures with identification of novel genetic profiles that may help clinicians in the prognosis of patients with ACC and potentially unraveling new diagnostic targets. Our findings, therefore, offer valuable insights that are not only pertinent to continued cancer research but also hold significant clinical implications.
