# Supplementary material for: Comprehensive genomic analysis of adrenocortical carcinoma reveals genetic profiles associated with patient survival
Source: ESMO Open. 2024 Jun 26;9(7):103617. doi: 10.1016/j.esmoop.2024.103617 (PMC11260375; doi:10.1016/j.esmoop.2024.103617)
Supplement: Supplementary Figures [file mmc1.pdf]

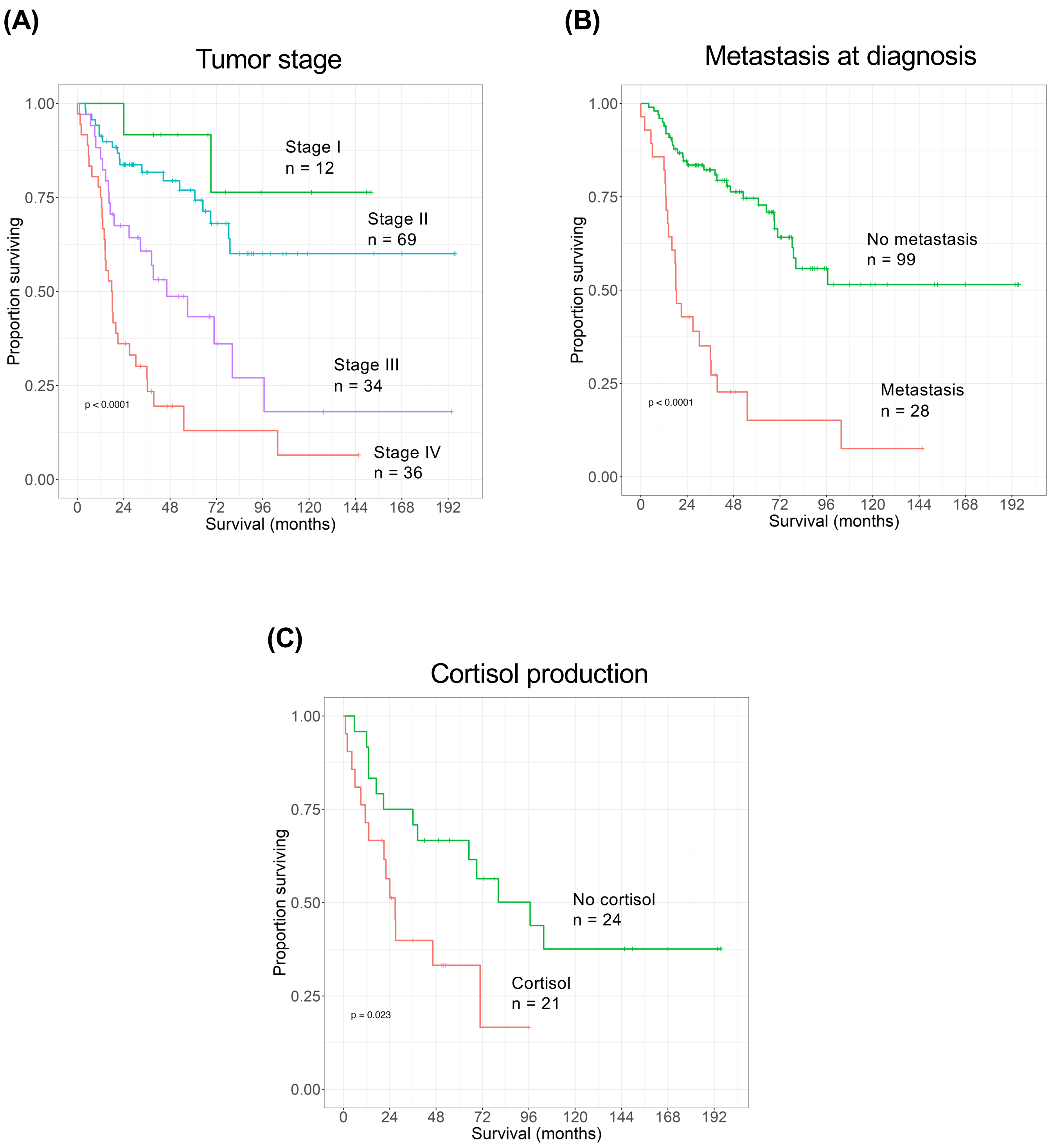

Supplementary Figure S1. Comparison of clinicopathological features with overall survival in patients with adrenocortical carcinoma in the YKD, TCGA and GEO cohorts combined. Inferior survival was associated with (A) higher tumor stage; (B) metastasis at diagnosis and (C) cortisol producing tumors. (A) and (B) uses data from the YKD and TCGA cohorts and (C) is based on data from the YKD and GEO cohorts.

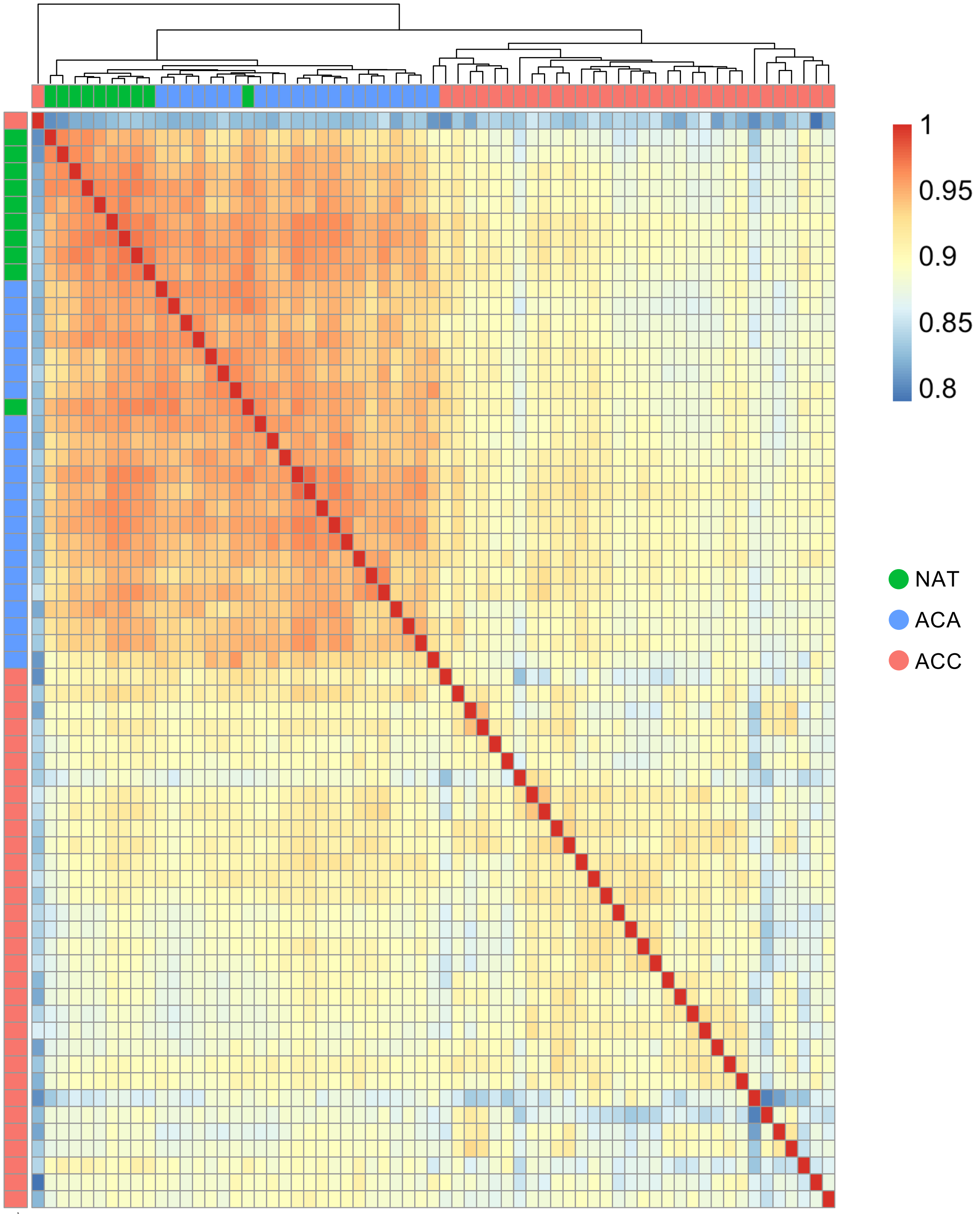

Supplementary Figure S2. Heatmap showing mRNA expression similarity between samples and categorized by tissue type from the GEO cohort. Adrenocortical carcinoma (ACC) showed higher heterogeneity compared to adrenocortical adenoma (ACA) and normal adrenal tissue (NAT) which showed closer similarity to each other. Identical samples had the same mRNA expression profiles which are red.
